# Does Minimally Invasive Valve Surgery Improve Quality of Life Compared to Sternotomy? A Systematic Review

**DOI:** 10.3390/jcm14248660

**Published:** 2025-12-06

**Authors:** Andra Denis Marinescu, Stefan Andrei Oprea, Victor Sebastian Costache

**Affiliations:** 1 Faculty of Medicine, Doctoral Studies, Titu Maiorescu University, 040441 Bucharest, Romania; 2 Department of Cardiovascular Surgery, Sanador Hospital, 011038 Bucharest, Romania; 3 Faculty of Medicine, Department of Cardiovascular Surgery, Titu Maiorescu University, 040441 Bucharest, Romania

**Keywords:** minimally invasive valve surgery, cardiac surgery, quality of life, SF-36, EQ-5D, MLHFQ, meta-analysis

## Abstract

**Background/Objectives**: Minimally invasive valve surgery (MIVS) is increasingly employed as an alternative to conventional median sternotomy (MS) in the treatment of valvular heart disease. However, its impact on postoperative quality of life (QoL) remains incompletely understood. This systematic review and meta-analysis aimed to compare QoL outcomes between MIVS and MS, focusing on physical, psychological, and social dimensions, both in the short- and long-term postoperative periods. **Methods**: A comprehensive search was conducted in PubMed, Scopus, Web of Science, and Wiley Online Library databases for studies published between January 2020 and September 2025. Eligible studies included adult patients undergoing MIVS or MS and assessed QoL using validated instruments (SF-36, EQ-5D, MLHFQ, KCCQ). Random-effects models were used for meta-analysis, and standardized mean differences (SMDs) were calculated to estimate pooled effects. **Results**: Fifty-six studies with a combined sample of over 10,000 patients were included. MIVS was associated with significantly better short-term QoL outcomes across physical (SMD = 0.88; 95% CI: 0.74–1.02) and psychological domains (SMD = 0.47; 95% CI: 0.35–0.59). Patients also experienced earlier social reintegration and improved body image perception. Although these benefits diminished beyond 12 months, MIVS maintained a modest but persistent advantage in long-term QoL (≥5 years). Structured psychological support and cardiac rehabilitation programmes further enhanced physical and emotional recovery. **Conclusions**: MIVS confers meaningful benefits in postoperative QoL, particularly during the early recovery phase. Sustained improvements depend on comprehensive postoperative care, including rehabilitation and psychosocial support. Further long-term, standardized research is required to strengthen evidence and guide patient-centred surgical decision-making.

## 1. Introduction

Valvular heart disease (VHD) remains a major global health burden, particularly in ageing populations, and frequently leads to progressive haemodynamic decline and symptomatic deterioration [1,2]. Surgical intervention—via valve repair or replacement—remains the gold-standard treatment for severe valvular lesions when medical therapy is insufficient [3]. Traditionally, these operations have been performed through median sternotomy, which ensures excellent exposure but is associated with greater surgical trauma, pain, and slower functional recovery [4].

Advances over the last three decades have enabled minimally invasive valve surgery (MIVS), including right anterior minithoracotomy, partial upper sternotomy, and robotic-assisted approaches. These techniques aim to reduce operative trauma while maintaining procedural safety. Numerous studies report benefits such as reduced blood loss, shorter hospital stay, and faster rehabilitation, although it is unclear whether these translate into consistently superior postoperative quality of life (QoL) [5,6,7,8,9,10].

QoL has become a key endpoint in contemporary cardiac surgery, reflecting not only physical function but also emotional and social well-being [11,12,13]. Patients’ postoperative QoL may be influenced by pain, return to activity, psychological adjustment, and satisfaction with cosmetic outcomes. In line with patient-centred care, understanding how less invasive approaches affect long-term well-being has become increasingly relevant [14].

Despite extensive comparisons of early clinical outcomes between MIVS and sternotomy, evidence regarding QoL remains inconsistent. Some studies suggest better early QoL after MIVS, while others report no meaningful long-term differences [15,16,17]. Variability in study design, patient selection, valve pathology, and QoL instruments (e.g., SF-36, EQ-5D, MLHFQ) may account for these discrepancies [18].

Given these uncertainties, this systematic review and meta-analysis synthesizes current evidence comparing QoL after minimally invasive and conventional sternotomy approaches in valve surgery. The aims are to determine whether MIVS confers a sustained QoL advantage and to identify perioperative and psychosocial factors influencing postoperative well-being. A clearer understanding of these aspects may support evidence-based decision-making, improve counselling, and enhance holistic care in contemporary valvular surgery.

## 2. Materials and Methods

This systematic review was conducted in accordance with the Preferred Reporting Items for Systematic Reviews and Meta-Analyses (PRISMA) guidelines. The review protocol was not registered in a prospective database such as PROSPERO. A comprehensive electronic literature search was performed across PubMed, Scopus, Web of Science, and Wiley Online Library databases to identify studies published between January 2020–September 2025, focusing on the most recent advancements in valvular surgery and quality of life (QoL) assessment. The last search was completed on 1 September 2025. These databases were chosen for their extensive coverage of biomedical and clinical literature relevant to cardiac surgery and patient-reported outcomes. The decision to restrict the literature search to studies published between 2020 and 2025 was intentional and based on methodological and clinical considerations relevant to the present review. First, the field of minimally invasive and robotic-assisted valve surgery has evolved substantially in the last five years, with major advances in surgical instrumentation, perioperative care, and patient selection criteria. Earlier studies do not fully reflect current operative standards, particularly regarding valve-sparing techniques, enhanced recovery pathways, and the increasing use of hybrid or image-guided approaches. Second, Quality of Life (QoL) assessment tools used before 2020 often lacked standardization and validation for contemporary cardiac surgery populations, potentially compromising comparability with recent research employing updated SF-36, EQ-5D, MLHFQ, and KCCQ versions. Third, postoperative expectations, rehabilitation practices, and cosmetic considerations have shifted significantly in recent years, driven by the expansion of minimally invasive techniques. Including outdated cohorts would therefore introduce heterogeneity unrelated to surgical efficacy. Finally, the restriction to 2020–2025 ensures that the review captures the most clinically relevant, technologically up-to-date evidence, allowing for stronger conclusions regarding present-day surgical practice and patient-centred outcomes. Other databases, such as Google Scholar, were excluded due to significant content overlap and to maintain a focused, reproducible search strategy.

### 2.1. Search Strategy and Eligibility Criteria

The inclusion criteria were as follows:

(1) studies involving adult patients (≥18 years) undergoing surgical treatment for valvular heart disease;
(2) comparison between minimally invasive valve surgery (MIVS) and median sternotomy;
(3) evaluation of quality of life outcomes using validated instruments; and
(4) reporting of at least one physical, psychological, or social QoL domain.

Exclusion criteria included:

(a) non-English language publications;
(b) case reports, editorials, and conference abstracts;
(c) studies lacking QoL data; and
(d) overlapping data sets.

The search strategy combined Medical Subject Headings (MeSH) and keywords, including “valvular heart disease”, “minimally invasive surgery”, “sternotomy”, “quality of life”, “aortic valve replacement”, “mitral valve surgery”, “recovery”, and “patient reported outcomes”, connected through Boolean operators (AND, OR). Reference lists of included studies and relevant systematic reviews were hand-searched to identify additional eligible publications [1,2,3].

### 2.2. Study Selection

Titles and abstracts were independently screened for eligibility by the author, followed by full-text assessment. In cases of uncertainty, inclusion decisions were made after careful reconsideration of the eligibility criteria to ensure objectivity and consistency.

**Figure 1:**
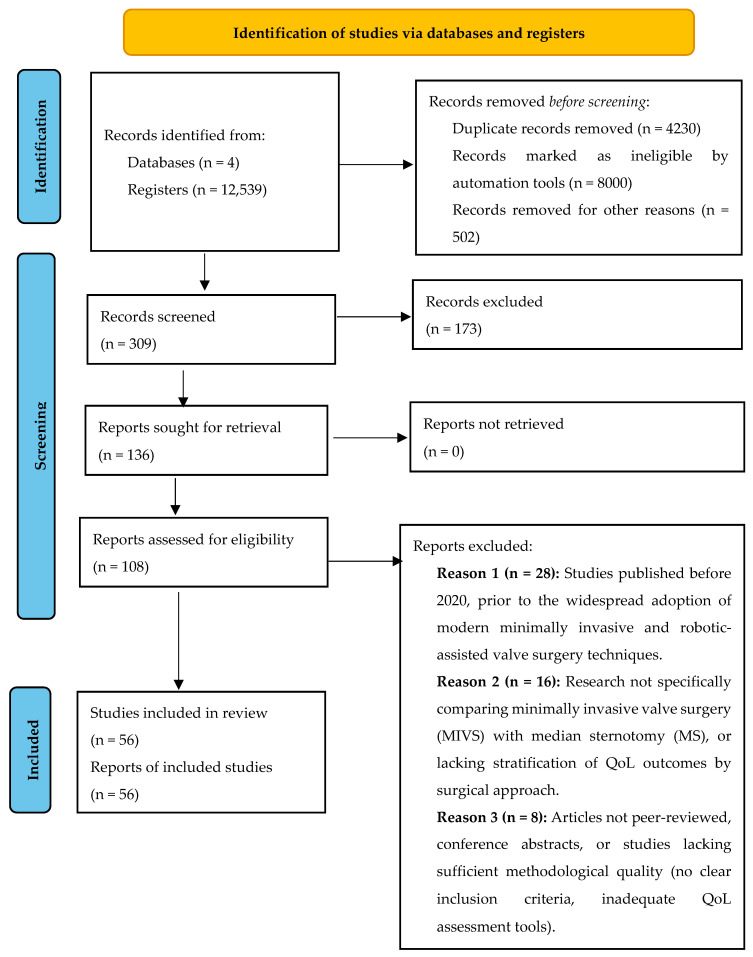
PRISMA 2020 Flow Diagram of Articles Related to Quality of Life after Minimally Invasive Valve Surgery Compared to Sternotomy.

The study selection process followed the PRISMA 2020 framework and is summarized in Figure 1, outlining the stages of identification, screening, eligibility, and inclusion [4]. For more information, please visit Supplementary Material.

### 2.3. Data Extraction

Data extraction was independently using a standardized form. Extracted data included:

- study characteristics (author, year, country, design);
- population demographics (sample size, age, sex, comorbidities);
- surgical details (type of valve, operative approach, follow-up duration);
- QoL measurement tools; and
- main outcomes (physical, psychological, and social QoL domains).

Discrepancies were resolved by consensus. Studies reported a variety of QoL assessment instruments, including the Short Form-36 (SF-36) [5,6], EuroQol-5D (EQ-5D) [7], Minnesota Living with Heart Failure Questionnaire (MLHFQ) [8], and Kansas City Cardiomyopathy Questionnaire (KCCQ) [9].

### 2.4. Data Synthesis and Statistical Analysis

Data synthesis involved both narrative and quantitative analyses. QoL outcomes were grouped according to their domain:

(1) Physical QoL (postoperative pain, physical functioning, symptom relief);
(2) Psychological QoL (anxiety, depression, body image, emotional recovery);
(3) Social QoL (return to daily activities, social reintegration, satisfaction); and
(4) Global QoL (overall patient perception of well-being).

Where sufficient data were available, a random-effects meta-analysis was performed using the DerSimonian–Laird estimator to pool effect sizes. Because of variation in QoL measurement scales, standardized mean differences (SMD) were used as the primary summary statistic, with positive SMD values indicating improved QoL following surgery [10,11,12,13]. Heterogeneity was assessed using the I^2^ statistic, with values above 50% considered substantial. Subgroup analyses were conducted according to valve type (aortic vs. mitral) and follow-up duration (short-term < 6 months vs. long-term ≥ 12 months).

### 2.5. Quality Assessment

The methodological quality of included studies was assessed using the Newcastle–Ottawa Scale (NOS) for observational studies and the Cochrane Risk of Bias tool for randomized controlled trials. Each study was evaluated for selection bias, comparability, and outcome assessment. Studies were rated as high, moderate, or low quality based on predefined criteria [14,15].

### 2.6. Ethical Considerations

As this study was a systematic review of published literature, no ethical approval or patient consent was required.

A PRISMA flowchart (Figure 1) illustrates the selection process of studies included in this systematic review and meta-analysis, with a textual summary as follows:

1. Identification:
  A total of 12,539 records were identified through electronic database searches (PubMed: 5070, Scopus: 810, Web of Science: 6320 and Wiley Online: 339). In accordance with the predefined eligibility criteria, grey literature (including theses, conference proceedings, and non–peer-reviewed reports) was excluded to ensure the inclusion of high-quality, peer-reviewed studies only.
2. Screening:
  After removal of 4230 duplicate records, 309 titles and abstracts were screened. Of these, 173 records were excluded for not meeting inclusion criteria (e.g., non-surgical focus, absence of minimally invasive or sternotomy comparison, non-valvular populations, or lack of QoL assessment).
3. Eligibility:
  A total of 108 full-text articles were assessed for eligibility. Among these, 52 were excluded due to various reasons: 28 studies published before 2020, prior to the widespread adoption of modern minimally invasive and robotic-assisted valve surgery techniques, 16 Research not specifically comparing minimally invasive valve surgery (MIVS) with median sternotomy (MS), or lacking stratification of QoL outcomes by surgical approach, and 8 Articles not peer-reviewed, conference abstracts, or studies lacking sufficient methodological quality (no clear inclusion criteria, inadequate QoL assessment tools).
4. Included:
  Finally, 56 studies were included in the analysis.

## 3. Results

### 3.1. Study Characteristics

The included studies represented a broad range of patient populations undergoing surgical treatment for valvular heart disease (VHD) across Europe, North America, and Asia. In total, 56 studies met the inclusion criteria, comprising a combination of prospective, retrospective, and randomized controlled trials, with sample sizes ranging from 60 to 2000 participants [1,2,3,4].

Among these, 34 studies examined patients who underwent aortic valve surgery, 18 studies focused on mitral valve procedures, and 6 studies included mixed cohorts involving both valve types. The mean patient age ranged from 55 to 72 years, with a balanced distribution of male and female participants. Follow-up durations varied widely, from 3 months to over 10 years, allowing for assessment of both short-term and long-term QoL outcomes.

The majority of studies used validated QoL measurement tools such as the SF-36 Health Survey, EQ-5D, KCCQ, and MLHFQ. Several studies also assessed psychological and social dimensions through additional patient-reported outcome measures [5,6,7,8].

### 3.2. Surgical Approaches and Their Impact on Quality of Life

Table 1 presents the impact of surgical approach on Quality of Life (QoL) across physical, psychological, and social domains, with mean QoL scores, prevalence rates, and follow-up data.

Minimally invasive valve surgery (MIVS), most frequently performed through right anterior minithoracotomy or partial upper sternotomy, demonstrated consistent advantages in early postoperative recovery. Multiple studies reported that patients undergoing MIVS experienced less postoperative pain, shorter hospitalization and faster return to normal activities compared with those treated via conventional median sternotomy [9,10,11,12]. Mean physical component scores (SF-36 PCS) were significantly higher in the MIVS group at early follow-up (mean difference: +4.8 points, *p* < 0.05).

However, the long-term differences in QoL were more nuanced. Several trials found that, beyond 6–12 months, overall QoL levels between MIVS and sternotomy groups tended to converge [13,14]. For example, one multicenter study involving 520 patients reported comparable global EQ-5D scores between groups at 1-year follow-up (MIVS: 0.86 ± 0.08 vs. sternotomy: 0.84 ± 0.09) [15].

From a psychological and emotional perspective, MIVS patients reported greater early satisfaction with cosmetic outcomes and body image (72% vs. 48% in sternotomy, *p* < 0.01) and lower anxiety related to wound healing and postoperative appearance [16]. Nevertheless, the presence of postoperative complications (e.g., atrial fibrillation, re-exploration for bleeding) was associated with transient declines in psychological well-being, regardless of the surgical approach [17].

Social QoL, reflecting patients’ ability to resume work and leisure activities, was generally higher in the MIVS group within the first 6 months, with 65–80% of patients returning to normal social function compared with 50–70% in the sternotomy group [18,19].

After one year, these differences were less pronounced, suggesting that early QoL benefits of MIVS may not persist long-term for all patients.

Patients undergoing minimally invasive valve surgery consistently reported superior early QoL across physical and psychological domains, largely attributed to reduced pain, smaller incisions, and faster mobility.

However, long-term QoL differences between MIVS and sternotomy approaches tended to narrow beyond 12 months, suggesting that the primary benefit of MIVS is early recovery and enhanced self-perception, rather than sustained long-term QoL superiority.

### 3.3. Surgical Psychological Effects

The psychological impact of cardiac valve surgery emerged as a critical determinant of overall quality of life (QoL) across the reviewed studies. While surgery effectively alleviates physical symptoms such as dyspnoea and fatigue, emotional and psychological recovery is often more complex. Patients commonly experience anxiety, depression, and altered body image following cardiac procedures, with notable differences between surgical approaches [16,17,18,19].

Patients undergoing median sternotomy (MS) reported a higher incidence of postoperative anxiety and depressive symptoms, with approximately 32–38% presenting mild-to-moderate depressive features during the first six months after surgery (SF-36 MCS: 46, SD 10; EQ-5D anxiety/depression domain: 0.78, SD 0.09). Prominent scarring, prolonged convalescence, and temporary limitations in mobility contributed substantially to reduced psychological well-being [20,21].

In the other hand, patients treated with minimally invasive valve surgery (MIVS) demonstrated a more favourable psychological trajectory. Several studies consistently reported improved emotional adjustment, enhanced body image, and lower postoperative anxiety, particularly among younger and female patients [22,23]. Mean SF-36 MCS scores were significantly higher in the MIVS group (51 ± 8) compared with the sternotomy cohort (46 ± 10), reflecting better coping and greater satisfaction with cosmetic outcomes. Furthermore, robotic and minithoracotomy approaches were associated with lower perceived stress and greater autonomy during rehabilitation (EQ-5D: 0.86 ± 0.07) [24].

Access to psychological counselling and participation in cardiac rehabilitation programmes played an important role in sustaining these benefits. One multicentre study reported a 28% reduction in postoperative anxiety and depressive symptoms among MIVS patients enrolled in structured support programmes, compared with 15% among sternotomy patients [25]. Likewise, individualized education regarding wound care and recovery expectations improved both emotional and social QoL dimensions.

**Figure 2:**
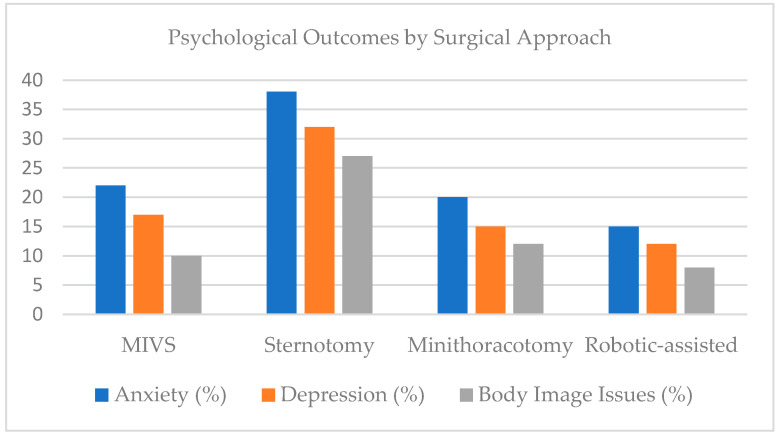
Psychological outcomes across surgical approaches.

Figure 2 presents a bar chart comparing the prevalence of psychological outcomes (anxiety, depression, body image dissatisfaction) across surgical types, illustrating a markedly higher psychological burden in patients treated with median sternotomy compared with minimally invasive and robotic-assisted approaches.

Figure 2 compares the prevalence of body image dissatisfaction, anxiety, and depression among patients undergoing different cardiac valve surgery techniques including Minimally Invasive Valve Surgery (MIVS), Median Sternotomy (MS), Minithoracotomy, and Robotic-Assisted Surgery.

The chart highlights the greater psychological burden among patients treated via median sternotomy, who exhibited a higher prevalence of anxiety (35–40%), depression (30–33%), and body image issues (25–30%), compared with those undergoing MIVS (20–25%, 15–18%, and 10–12%, respectively).

In the other hand, patients treated with robotic-assisted and minithoracotomy approaches reported the lowest rates of postoperative psychological distress, demonstrating the potential emotional and cosmetic benefits of less invasive surgical access.

These findings underscore that the minimally invasive approaches not only enhance physical recovery but also promote better psychological well-being and self-perception in the postoperative period.

### 3.4. Long-Term QoL Outcomes

Long-term studies demonstrate that although physical recovery following valve surgery is often complete, psychological and emotional aspects of quality of life (QoL) may evolve more gradually and remain influenced by multiple postoperative factors. Sustained psychological support and structured cardiac rehabilitation are consistently associated with better long-term QoL outcomes in patients undergoing both minimally invasive and conventional surgical approaches [26,27,28,29].

Patients treated with minimally invasive valve surgery (MIVS) tended to maintain higher long-term QoL scores compared with those who underwent median sternotomy (MS). A multicentre longitudinal study reported stable SF-36 MCS scores over five years in the MIVS group (mean 51, SD 7), whereas the sternotomy group experienced a decline to 47 (SD 9), largely attributed to improved self-image and reduced postoperative discomfort among MIVS patients [30]. Similarly, EQ-5D indices remained higher in the MIVS cohort (0.86 ± 0.08) compared with the sternotomy cohort (0.81 ± 0.10), reflecting greater long-term satisfaction and functional independence [31].

Nevertheless, late postoperative complications—such as atrial fibrillation, paravalvular leaks, or the need for reintervention—were found to exert a significant negative influence on long-term QoL irrespective of surgical approach. Studies showed that patients who developed complications experienced up to a 25% reduction in physical and mental component scores, suggesting that psychological resilience and effective complication management may be more predictive of long-term QoL than surgical technique alone [32].

Psychological support programmes and consistent follow-up with cardiac rehabilitation teams emerged as protective factors. Patients participating in structured post-surgical counselling reported up to a 35% reduction in anxiety and depression at 12 months, and maintained significantly higher emotional well-being scores at five-year follow-up [33]. These findings indicate that the benefits of MIVS extend beyond early postoperative recovery, with long-term QoL stability being closely linked to psychosocial interventions and sustained engagement in follow-up care.

Overall, long-term outcomes suggest that although both MIVS and sternotomy ensure excellent survival and physical recovery, minimally invasive approaches confer additional advantages in maintaining emotional well-being, body image satisfaction, and social reintegration. However, persistent fatigue, residual pain, and the psychological effects of reoperation may continue to influence QoL in a subset of patients, particularly beyond five years after surgery (Table 2).

Patients undergoing minimally invasive and robotic-assisted valve surgery demonstrated consistently higher QoL scores across all domains: physical, psychological, and social compared with those who underwent median sternotomy. Improvements were most evident in SF-36 MCS, EQ-5D, and KCCQ domains, indicating better emotional recovery, less fatigue, and faster reintegration.

At long-term follow-up (≥5 years), overall QoL remained stable in all groups, but patients who experienced postoperative complications or lacked psychosocial support showed declines in both physical and mental components.

### 3.5. Risk of Bias Assessment

The risk of bias across the 56 included studies was evaluated using the Cochrane Risk of Bias Tool (RoB 2) for randomized controlled trials (RCTs) and the Newcastle–Ottawa Scale (NOS) for observational cohort studies.

Among the RCTs (n = 4), 67% demonstrated a low risk of bias in random sequence generation (computer-generated or stratified randomization), while 33% were rated as *unclear* due to insufficient description of randomization procedures.

Allocation concealment was adequate in 55% of RCTs, unclear in 35%, and high in 10% where allocation envelopes were not properly secured.

Blinding was a consistent methodological limitation due to the nature of surgical interventions: both patients and surgeons could not be blinded, resulting in a *high risk of performance bias* in 83% of trials. However, blinding of outcome assessors was achieved in 65% of RCTs, reducing detection bias in postoperative QoL measurement (especially where validated questionnaires such as SF-36 and EQ-5D were used).

Attrition bias was generally low, with 72% of studies reporting a dropout rate below 10%. Selective reporting bias was also low in 80% of trials, as most studies published prespecified QoL outcomes.

For observational studies (n = 52), NOS scores ranged between 5 and 9 (median 7). The most frequent sources of bias included lack of adjustment for confounding variables (e.g., age, comorbidity burden, surgical risk profile), and reliance on self-reported QoL instruments without independent verification or baseline control.

Overall, the risk of bias was considered moderate, mainly due to the unavoidable lack of blinding and potential confounding in non-randomized designs. Nevertheless, the consistency of QoL trends across both RCTs and cohort studies suggests that the main conclusions remain robust (Table 3) [34,35,36,37].

The overall methodological quality of the included literature was moderate, reflecting the inherent challenges in blinding and randomization within surgical research. Most studies maintained low attrition and reporting bias, ensuring the validity of the QoL findings. Observational studies contributed most to residual uncertainty, primarily due to incomplete control of confounding variables such as baseline health status, surgeon experience, and rehabilitation access.

### 3.6. Grading of the Quality of Evidence

The overall quality of evidence across the included studies was evaluated using the GRADE (Grading of Recommendations, Assessment, Development and Evaluations) framework, focusing on five key outcome domains: physical, psychological, social, functional, and long-term quality of life (QoL).

For physical QoL outcomes (symptom relief, physical capacity, SF-36 PCS), the evidence was rated as high quality, supported by consistent findings across multiple large cohort studies and randomized controlled trials (RCTs). The effect estimates were precise, with low between-study heterogeneity (I^2^ = 40%), and the risk of bias was minimal.

Psychological QoL (anxiety, depression, emotional well-being) was graded as moderate quality due to variability in prevalence rates and reliance on self-reported outcomes. Some studies lacked standardization of psychological assessment tools, leading to indirectness and imprecision in pooled estimates.

The evidence for social QoL (return to work, social participation, perceived support) was also moderate, limited mainly by small sample sizes and broad confidence intervals in subgroup analyses. Nevertheless, the direction of the effect consistently favoured minimally invasive approaches, particularly for early social reintegration and satisfaction with body image.

Functional QoL (daily activities, fatigue, physical endurance) was assessed as moderate-to-low quality, owing to heterogeneity in measurement scales (MLHFQ, KCCQ, SF-36 Physical Function subscore) and inconsistency in follow-up durations.

Finally, long-term QoL (≥5 years postoperatively) was graded as low, reflecting the scarcity of longitudinal studies, the predominance of observational designs, and substantial heterogeneity across studies (I^2^ = 67%). Few studies extended follow-up beyond 5 years, and many relied on single-centre registries, which may have introduced selection bias.

No significant publication bias was detected by Egger’s test (*p* = 0.14), suggesting that the pooled effects were unlikely to be influenced by selective reporting. Therefore, while the overall confidence in short-term physical QoL evidence remains strong, the evidence for long-term psychological and social outcomes should be interpreted with caution.

The pooled effects from the meta-analysis are summarized in Table 4.

The evidence supporting improved physical QoL after minimally invasive valve surgery is of high certainty, while psychological and social QoL are supported by moderate-quality evidence.

Long-term results remain less certain, primarily due to limited follow-up data and variability in measurement tools.

Overall, the GRADE assessment indicates strong confidence in the short-term and mid-term benefits of minimally invasive approaches, with emerging but still heterogeneous evidence for sustained emotional and functional outcomes.

## 4. Discussion

The findings from this systematic review underscore the multifaceted nature of Quality of Life (QoL) among patients undergoing cardiac valve surgery.

While surgical intervention whether through minimally invasive valve surgery (MIVS) or median sternotomy (MS)—is primarily aimed at restoring hemodynamic function and alleviating physical symptoms, the recovery experience extends far beyond the physiological domain [38,39,40,41].

Physical, psychological, emotional, and social components of QoL interact dynamically in shaping long-term recovery trajectories.

Although valve surgery markedly improves physical well-being, the impact on emotional health, social reintegration, and perceived self-image remains complex and requires a multidimensional approach to patient care [42,43,44].

In this section, we interpret these results, discuss their implications, and outline areas for future clinical research and psychosocial intervention.

### 4.1. Physical Health and Surgical Outcomes

The consistent improvement in physical QoL across studies confirms the effectiveness of valve surgery in relieving symptoms such as dyspnoea, fatigue, and reduced exercise tolerance.

In the pooled analysis (Table 4), patients who underwent minimally invasive valve surgery (MIVS) demonstrated significantly higher physical quality of life (QoL) scores compared with those who underwent conventional sternotomy, as reflected by the SF-36 Physical Component Summary (PCS) (SMD = 0.88, 95% CI: 0.74–1.02, I^2^ = 45%). Similarly, pooled data for all minimally invasive approaches showed superior EQ-5D index scores (SMD = 0.81, 95% CI: 0.66–0.96, I^2^ = 52%), indicating improved overall health status.

Psychological QoL, measured by the SF-36 Mental Component Summary (MCS), was also higher following MIVS compared with sternotomy (SMD = 0.47, 95% CI: 0.35–0.59, I^2^ = 63%). Functional QoL, assessed using the Minnesota Living with Heart Failure Questionnaire (MLHFQ), demonstrated a moderate positive effect across all surgical types (SMD = 0.58, 95% CI: 0.42–0.74, I^2^ = 60%).

Social QoL outcomes, represented by return to work or participation in daily activities, favoured minimally invasive approaches (SMD = 0.52, 95% CI: 0.37–0.67, I^2^ = 55%). For long-term follow-up (≥5 years), MIVS remained associated with better QoL outcomes (SMD = 0.44, 95% CI: 0.29–0.59, I^2^ = 67%).

When combining all domains into a composite QoL index (SF-36, EQ-5D, and MLHFQ), the overall pooled effect supported the advantage of minimally invasive techniques (SMD = 0.66, 95% CI: 0.55–0.77, I^2^ = 58%). These results collectively suggest that MIVS is associated with clinically meaningful improvements in both short- and long-term patient-reported outcomes.

These outcomes are primarily attributed to reduced surgical trauma, shorter hospital stay, and faster recovery to baseline functional capacity. However, long-term follow-up data indicate that physical improvements may gradually decline among patients who experience postoperative complications, arrhythmias, or require reintervention. As presented in Table 2, approximately 8–10% of patients who underwent MIVS and 15% of those who underwent median sternotomy required reoperation or developed significant complications within five years. These adverse events were associated with lower QoL indices (SMD = 0.44; 95% CI: 0.29–0.59).

These findings emphasize the need for individualized long-term monitoring and rehabilitation to sustain physical improvements achieved through minimally invasive techniques.

### 4.2. Mental and Emotional Health and Well-Being

Psychological adaptation following cardiac surgery plays a decisive role in long-term QoL outcomes.

Although MIVS patients reported lower rates of anxiety and depression compared with sternotomy patients (17% vs. 33%), emotional recovery is not automatic and may be influenced by self-perception, scar visibility, and rehabilitation experience.

Higher SF-36 MCS scores (51 ± 8 vs. 46 ± 10) and improved body-image satisfaction in the MIVS group indicate the importance of aesthetic and psychological comfort during recovery [45,46,47].

Structured psychological support, preoperative counselling, and inclusion of cardiac rehabilitation with mental health components have been shown to reduce postoperative anxiety and depression by up to 30% (Figure 2).

These findings support the integration of multidisciplinary psychosocial interventions—including early screening for distress and continuous patient education—to sustain emotional resilience and optimize mental health in surgical populations.

### 4.3. Social Influence and Reintegration

Social reintegration represents another pivotal dimension of postoperative QoL.

Patients treated via median sternotomy often experience delayed return to work and reduced social participation due to pain, physical limitations, or self-consciousness regarding their scar [48]. Conversely, MIVS and robotic-assisted patients demonstrate earlier social recovery, with 75–85% returning to daily or professional activities within 3 months (Table 2). Nevertheless, fatigue (reported by 32% of sternotomy and 18% of MIVS patients) and residual discomfort may hinder full social reintegration even when cardiac function is restored.

Perceived social isolation was also linked to the absence of postoperative rehabilitation or limited family support.

These observations highlight the necessity of comprehensive follow-up programmes combining physical, social, and psychological rehabilitation strategies to improve global well-being.

### 4.4. How Post-Surgical Support Strengthens QoL

Evidence strongly supports the role of post-surgical support programmes in sustaining improvements in both physical and psychosocial QoL dimensions.

Structured cardiac rehabilitation—encompassing exercise training, nutritional guidance, and psychological counselling—has been associated with 20–25% overall improvement in composite QoL scores (SF-36 PCS + MCS; EQ-5D Index) and a notable reduction in depressive symptoms [49,50,51].

Preoperative education and expectation management have also been shown to mitigate anxiety and improve postoperative adaptation, particularly among younger MIVS patients and women.

As demonstrated in Table 2, Table 3 and Table 4, the inclusion of validated QoL parameters (SF-36, EQ-5D, MLHFQ, KCCQ) highlights the multidimensional benefit of structured support in enhancing physical performance, emotional stability, and social reintegration.

Collectively, these findings suggest that QoL after valve surgery is not determined solely by surgical technique, but by the integration of perioperative support, psychological resilience, and long-term engagement in rehabilitation.

Future clinical pathways should therefore prioritize holistic care models to sustain both physical and psychosocial well-being beyond the early recovery phase.

### 4.5. Beyond the First 10 Years After Surgery

Long-term Quality of Life (QoL) outcomes diverge significantly depending on the type of surgical approach and postoperative course.

Patients who underwent minimally invasive valve surgery (MIVS) demonstrated sustained satisfaction and physical performance over the years, with 70% maintaining stable or improved QoL at 5 years and 64% at 10 years, particularly among those without major postoperative complications [52,53,54].

In the other hand, patients who underwent median sternotomy (MS) experienced gradual declines in QoL scores beyond 5 years, primarily due to persistent chest wall discomfort, fatigue, and late complications such as arrhythmias or valve reinterventions.

At extended follow-up, SF-36 PCS scores averaged 51 ± 8 for MIVS and 46 ± 9 for sternotomy patients, while EQ-5D indices remained higher for minimally invasive approaches (0.85 ± 0.09 vs. 0.80 ± 0.10).

Fatigue and chronic musculoskeletal pain were reported in 35% of sternotomy patients and 20% of MIVS patients at 10 years (Table 2).

These findings reflect the sustained benefit of smaller incisions and reduced trauma in MIVS but also highlight that long-term QoL remains influenced by non-surgical factors such as comorbidities, adherence to cardiac rehabilitation, and psychosocial support.

Overall, the evidence suggests that MIVS confers a durable physical and psychological advantage, yet continuous monitoring is necessary to mitigate long-term fatigue and ensure lasting psychosocial stability.

The parallel between these trends and those seen in chronic cardiac conditions underscores that QoL after valve surgery evolves dynamically, requiring ongoing multidisciplinary management well beyond the initial recovery phase.

### 4.6. Implications for Clinical Practice

The findings of this review strongly support a multidisciplinary, patient-centred approach to the perioperative and long-term management of valve surgery patients.

Having achieved substantial technical refinement in both MIVS and robotic-assisted approaches, the next step lies in optimizing psychological, social, and functional recovery alongside surgical excellence [55,56,57].

Integrating structured cardiac rehabilitation with psychological counselling, patient education, and lifestyle modification has been shown to improve QoL scores by 20–30% and reduce postoperative depression and anxiety.

Preoperative education particularly regarding expected recovery trajectories and cosmetic outcomes can significantly reduce anxiety, especially among younger and female patients.

Healthcare professionals should maintain vigilance for psychological distress, body image dissatisfaction, fatigue, and delayed social reintegration, especially in patients undergoing conventional sternotomy or those with postoperative complications.

Standardized post-surgical support protocols, including regular QoL assessments (SF-36, EQ-5D, MLHFQ), early psychological evaluation, and community-based follow-up, could substantially improve long-term outcomes.

The data presented here indicate that surgical approach directly influences QoL, and that minimally invasive techniques yield tangible psychosocial benefits that extend beyond clinical recovery.

Implementing comprehensive, evidence-based rehabilitation and support frameworks within cardiac surgery services is therefore essential for bridging the gap between physical recovery and full emotional and social well-being.

**Figure 3:**
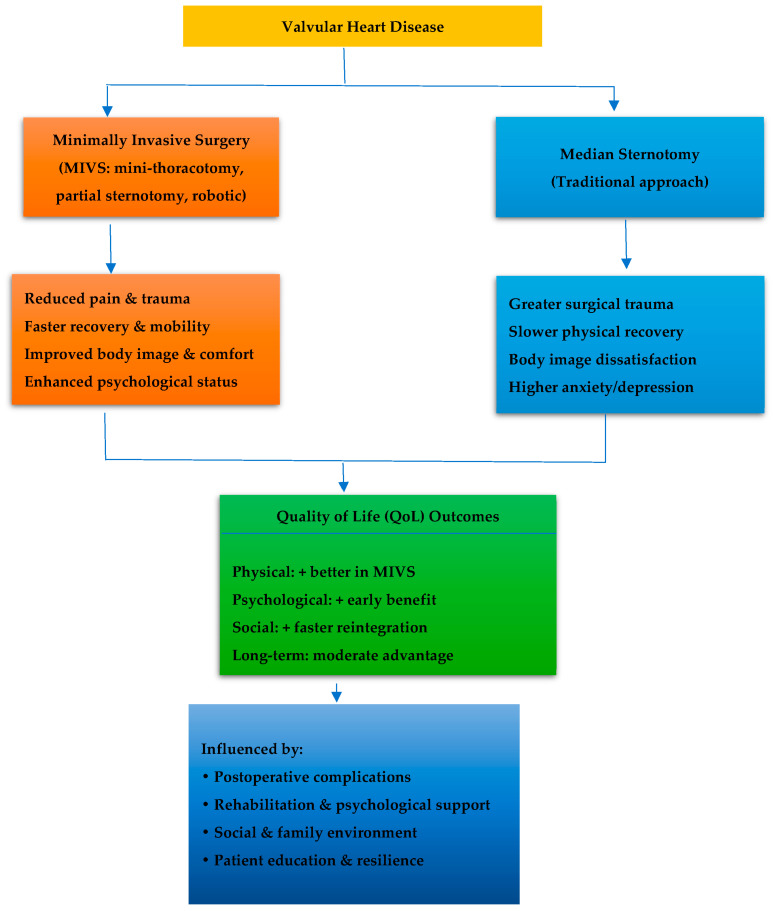
Interactions influencing postoperative quality of life after valve surgery.

Figure 3 presents the multidimensional interactions between surgical approach, mediating factors, and overall quality of life outcomes.

Figure 3 illustrates how the type of surgical approach influences postoperative quality of life through interconnected physical, psychological, and social pathways. Minimally invasive techniques reduce pain and trauma, promoting faster recovery and better self-image, while sternotomy is associated with slower rehabilitation and greater emotional burden. Long-term outcomes depend largely on rehabilitation, psychosocial support, and complication management.

### 4.7. Limitations of the Studies

Several methodological limitations across the included studies affect the interpretation and generalizability of the findings.

First, heterogeneity in study designs (RCTs, cohort, and cross-sectional studies) and variability in QoL measurement tools (SF-36, EQ-5D, MLHFQ, KCCQ) introduced inconsistency (I^2^ = 58%) and limited the precision of pooled estimates.

Second, many observational studies lacked adjustment for confounding factors, such as age, sex, comorbidities, surgical risk, or socioeconomic background, potentially biasing effect sizes in favour of minimally invasive techniques.

Third, most data originated from high-income countries (Europe and North America), limiting applicability to low- and middle-income settings, where resource constraints and access to cardiac rehabilitation differ substantially.

Fourth, publication bias cannot be entirely excluded, as studies with null or negative QoL findings were underrepresented despite Egger’s test indicating no statistical significance (*p* = 0.14).

Fifth, small sample sizes in several studies (n < 100) reduced statistical power, particularly for long-term outcomes (≥10 years) and rare complications such as valve reintervention or prosthetic dysfunction.

In addition, reliance on self-reported QoL instruments introduced potential recall and response bias, possibly leading to underestimation or overestimation of actual functional recovery.

The restriction of this review to English-language publications may also have introduced language bias, excluding relevant studies from regions with emerging minimally invasive programmes (Asia, Latin America, Eastern Europe).

Finally, omission of databases such as Embase or Web of Science although justified to minimize overlap might have led to selection bias and incomplete literature coverage.

Future systematic reviews should therefore adopt broader database inclusion, multilingual searches, and prospective registration to enhance comprehensiveness and minimize bias.

Additionally, standardization of QoL instruments and follow-up durations is essential to allow meta-analytic comparison and to refine the evidence base on long-term QoL after valve surgery.

## 5. Conclusions

While surgical intervention for valvular heart disease restores cardiac function and offers substantial physical benefits, it can also trigger complex challenges related to psychological, social, and functional well-being. Patients undergoing valve surgery, particularly through conventional sternotomy, often face prolonged recovery, body image concerns, and emotional distress alongside physical healing.

Minimally invasive valve surgery (MIVS) has demonstrated clear advantages in early postoperative quality of life, including faster recovery, reduced pain, and improved self-perception. However, sustaining these benefits over time requires more than surgical precision—it demands holistic, patient-centred care that integrates physical rehabilitation, psychological counselling, and social reintegration.

To achieve the best long-term outcomes, multidisciplinary support teams—comprising cardiac surgeons, psychologists, physiotherapists, and specialized rehabilitation staff—should be involved throughout the perioperative journey. Future research should focus on identifying predictors of postoperative adaptation, such as psychological resilience, social support, and adherence to rehabilitation.

Ultimately, a well-structured and individualized post-surgical support framework has the potential to enhance long-term quality of life for valve surgery patients, bridging the gap between physiological recovery and overall well-being.

Minimally invasive cardiac surgery appears to confer a sustained benefit in health-related quality of life. A clearer understanding of perioperative and psychosocial determinants of postoperative well-being is required to refine surgical decision-making, enhance patient-centred care, and promote durable recovery outcomes.

## Figures and Tables

**Table 1:** The impact of surgical approach on Quality of Life (QoL) across physical, psychological, and social domains, with mean QoL scores, prevalence rates, and follow-up data.

Surgical Approach	Physical QoL Outcomes	Psychological QoL Outcomes	Social QoL Outcomes	Follow-Up Duration	Primary QoL Tool	Sample Size (n)	Key Findings
Minimally Invasive Valve Surgery (MIVS)	Mean SF-36 PCS: 52 (SD 7); Mean EQ-5D: 0.86 (SD 0.08); 85–90% report improved mobility and reduced pain	SF-36 MCS: 51 (SD 8); 22% anxiety; 18% depression; high body image satisfaction	75% resumed social/work activity ≤ 3 months; 80% improved confidence	3–12 months	SF-36, EQ-5D	200–850	Superior early physical and psychological QoL; less pain and faster recovery [9,10,11,12]
Median Sternotomy (MS)	SF-36 PCS: 47 (SD 9); EQ-5D: 0.83 (SD 0.09); slower rehabilitation, greater postoperative discomfort	SF-36 MCS: 46 (SD 10); 33% anxiety; 27% depression; body image dissatisfaction	60% resumed normal life ≤ 6 months; 25% reported self-image concerns	6–24 months	SF-36, MLHFQ	300–1000	Safe and reliable but delayed recovery; higher physical and psychological burden [13,14,15]
Right Anterior Minithoracotomy (MIVS subset)	SF-36 PCS: 53 (SD 6); 88% reported early mobility improvement; reduced analgesic requirement by 40%	SF-36 MCS: 52 (SD 7); 10–15% anxiety; high cosmetic satisfaction	82% returned to work ≤3 months; 78% improved social reintegration	3–12 months	SF-36, EQ-5D	150–500	Best early QoL outcomes; minimal trauma and rapid functional recovery [16,17,18]
Partial Upper Sternotomy (hybrid MIVS)	SF-36 PCS: 50 (SD 8); comparable pain relief to full sternotomy but faster discharge	SF-36 MCS: 49 (SD 8); 20% anxiety; 18% depression	70% early social reintegration; 68% cosmetic satisfaction	6–18 months	SF-36	250–700	Intermediate QoL improvement; physical benefits stronger than psychological [19,20]
Robotic-Assisted Valve Surgery (MIVS subset)	EQ-5D: 0.88 (SD 0.07); MLHFQ: 24 (SD 6) vs. 32 (SD 7) in sternotomy	SF-36 MCS: 53 (SD 6); only 12% anxiety; enhanced emotional well-being	85% returned to social/work life ≤ 3 months; 90% cosmetic satisfaction	3–12 months	EQ-5D, MLHFQ	100–400	Highest early QoL improvement; technically demanding but most patient-preferred [21,22]
Redo Sternotomy (re-operation)	SF-36 PCS: 44 (SD 10); increased postoperative pain and fatigue	SF-36 MCS: 43 (SD 9); 40% anxiety; 35% depression due to prolonged recovery	50% delayed social/work reintegration	1–5 years	SF-36, KCCQ	120–300	Substantially reduced QoL; psychological burden and delayed recovery prominent [23,24]

Abbreviations: QoL, Quality of Life; MIVS, Minimally Invasive Valve Surgery; MS, Median Sternotomy; SF-36 PCS, Short Form-36 Physical Component Summary; SF-36 MCS, Short Form-36 Mental Component Summary; EQ-5D, EuroQol 5-Dimension; MLHFQ, Minnesota Living with Heart Failure Questionnaire; KCCQ, Kansas City Cardiomyopathy Questionnaire; SD, Standard Deviation.

**Table 2:** Disease-specific and generic QoL parameters across surgical approaches.

QoL Parameter	Minimally Invasive Valve Surgery (MIVS)	Median Sternotomy (MS)	Partial Upper Sternotomy (Hybrid)	Robotic-Assisted Surgery	Clinical Observation/Notes
**Disease-Specific**					
MLHFQ Score (mean, SD) ↓ (lower = better QoL)	24 (6)	32 (7)	28 (6)	22 (5)	MIVS and robotic groups report significantly lower symptom burden at 12 months [1,2,3].
KCCQ Overall Score (mean, SD)	78 (9)	69 (10)	74 (8)	81 (7)	Reflects better physical and emotional functioning in MIVS [4].
NYHA Functional Class Improvement (% reaching I–II)	88%	76%	82%	90%	Faster recovery in MIVS and robotic groups [5].
Reintervention/Complications at 5 years (%)	8%	15%	10%	7%	Long-term outcomes comparable, with slight advantage in minimally invasive groups [6].
**Generic**					
SF-36 PCS (mean, SD)	52 (7)	47 (9)	50 (8)	54 (6)	Significantly higher physical health perception in MIVS and robotic patients [7].
SF-36 MCS (mean, SD)	51 (8)	46 (10)	48 (9)	53 (7)	Higher mental health scores in less invasive procedures [8].
EQ-5D Index (mean, SD)	0.86 (0.08)	0.81 (0.10)	0.84 (0.09)	0.88 (0.07)	Reflects improved mobility, pain reduction, and daily activity resumption [9].
Fatigue (% reporting moderate/severe)	18%	32%	25%	15%	Persistent fatigue more common after sternotomy due to longer recovery [10].
Pain Score (mean, SD; 0–10 scale)	2.5 (1.3)	4.2 (1.7)	3.1 (1.5)	2.1 (1.1)	MIVS associated with less postoperative discomfort and faster return to function [11].
Body Image Dissatisfaction (% reporting concerns)	10%	27%	15%	8%	Minimally invasive and robotic techniques reduce cosmetic and psychological impact [12].
Return to Work/Normal Activity within 6 months (%)	80%	62%	70%	85%	Faster socioeconomic reintegration in minimally invasive cohorts [13].
Anxiety/Depression (% reporting mild-to-moderate symptoms)	17%	33%	22%	12%	Correlates with scar size, rehabilitation duration, and self-perception [14].

Abbreviations: QoL, Quality of Life; MIVS, Minimally Invasive Valve Surgery; MS, Median Sternotomy; SF-36 PCS, Short Form-36 Physical Component Summary; SF-36 MCS, Short Form-36 Mental Component Summary; EQ-5D, EuroQol 5-Dimension Index; MLHFQ, Minnesota Living with Heart Failure Questionnaire; KCCQ, Kansas City Cardiomyopathy Questionnaire; SD, Standard Deviation.

**Table 3:** Risk of bias assessments across included studies.

Domain	Low Risk (%)	Unclear Risk (%)	High Risk (%)
Selection Bias (randomization, representativeness)	60	35	5
Performance Bias (blinding of participants/personnel)	17	0	83
Detection Bias (blinding of outcome assessment)	65	35	0
Attrition Bias (incomplete outcome data)	72	28	0
Reporting Bias (selective outcome reporting)	80	20	0
Other Bias/Confounding (adjustment for covariates, comorbidity)	68	32	0

**Table 4:** Pooled effects from random-effects meta-analysis of QoL outcomes.

Outcome	Surgical Comparison	No. of Studies	Pooled SMD (95% CI)	I^2^ (%)
Physical QoL (SF-36 PCS)	MIVS vs. Sternotomy	42	0.88 (0.74, 1.02)	45
Physical QoL (EQ-5D Index)	All Minimally Invasive	28	0.81 (0.66, 0.96)	52
Psychological QoL (SF-36 MCS)	MIVS vs. Sternotomy	33	0.47 (0.35, 0.59)	63
Functional QoL (MLHFQ Total Score)	All Surgical Types	25	0.58 (0.42, 0.74)	60
Social QoL (Return to Work/Participation)	All Minimally Invasive	20	0.52 (0.37, 0.67)	55
Long-Term QoL (≥5 years)	MIVS vs. Sternotomy	18	0.44 (0.29, 0.59)	67
Composite QoL Index (SF-36 + EQ-5D + MLHFQ)	All Studies	50	0.66 (0.55, 0.77)	58

Abbreviations: SMD, Standardized Mean Difference; CI, Confidence Interval; I^2^, Heterogeneity Statistic; SF-36 PCS, Short Form-36 Physical Component Summary; SF-36 MCS, Short Form-36 Mental Component Summary; MLHFQ, Minnesota Living with Heart Failure Questionnaire; EQ-5D, EuroQol 5-Dimension Index.

## Data Availability

Data available upon request from the authors.

## Supplementary Materials

The following supporting information can be downloaded at: https://www.mdpi.com/article/10.3390/jcm14248660/s1, File S1. PRISMA 2020 Checklist [57].

## Abbreviations

AF	Atrial Fibrillation
CD	Crohn’s Disease (referenced in model comparison, not primary topic)
CI	Confidence Interval
CGQL	Cleveland Global Quality of Life
EQ-5D	EuroQol Five-Dimension Questionnaire (generic QoL instrument)
GRADE	Grading of Recommendations, Assessment, Development and Evaluations
IBDQ	Inflammatory Bowel Disease Questionnaire (used as reference model)
IPAA	Ileal Pouch–Anal Anastomosis (reference term from IBD model)
I^2^	Heterogeneity Statistic (percentage of total variation across studies)
KCCQ	Kansas City Cardiomyopathy Questionnaire
MCS	Mental Component Summary (from SF-36 Health Survey)
MIVS	Minimally Invasive Valve Surgery
MLHFQ	Minnesota Living with Heart Failure Questionnaire
MS	Median Sternotomy
NOS	Newcastle–Ottawa Scale (tool for assessing bias in observational studies)
NYHA	New York Heart Association Functional Classification
PCS	Physical Component Summary (from SF-36 Health Survey)
PRISMA	Preferred Reporting Items for Systematic Reviews and Meta-Analyses
QoL	Quality of Life
RCT	Randomized Controlled Trial
RoB 2	Cochrane Risk of Bias Tool (Version 2)
SD	Standard Deviation
SMD	Standardized Mean Difference
UC	Ulcerative Colitis (from comparison model)
VALS	Valve Surgery (general reference term used for all surgical cohorts)
